# The effects of implementing an electronic medical record on prehabilitation exercise prescription reporting for autologous stem cell transplantation

**DOI:** 10.1007/s00520-026-11421-3

**Published:** 2026-11-06

**Authors:** Jess Crowe, Shaza Abo, Linda Denehy, Amit Khot, Bernd Faveere, Samantha Hui Bon Hoa, Lucas Elias, Michelle Flechas, Marilyn Webster, Lara Edbrooke

**Affiliations:** 1 Physiotherapy & Occupational Therapy Department, Peter MacCallum Cancer Centre, Melbourne, Victoria Australia; 2 Department of Physiotherapy, The University of Melbourne, Melbourne, Victoria Australia; 3 Centre for Health Services Research in Cancer, Peter MacCallum Cancer Centre, Melbourne, Victoria Australia; 4 Department of Anaesthetics, Pain Medicine and Perioperative Medicine, Peter MacCallum Cancer Centre, Melbourne, Victoria Australia; 5 The Sir Peter MacCallum Department of Oncology, The University of Melbourne, Melbourne, Victoria Australia; 6 Department of Clinical Haematology, Peter MacCallum Cancer Centre and Royal Melbourne Hospital, Melbourne, Victoria Australia

**Keywords:** Prehabilitation, Exercise prescription, Implementation, Electronic medical record, Autologous stem cell transplant, Clinical documentation

## Abstract

**Purpose:**

This study evaluated the effect of implementating an electronic medical record (EMR) and tailored exercise case report form (CRF) on exercise prescription documentation in a prehabilitation service for patients with cancer prior to autologous stem-cell transplant. Specific aims were to evaluate documentation of exercise parameters — frequency, intensity, time, type (FITT) — and reasons for missing data; and compare findings to a pre-EMR implementation evaluation.

**Methods:**

A retrospective service evaluation using prospectively collected data from February 2023 to February 2024, reported according to STROBE guidelines. The implementation element of the RE-AIM framework was used for evaluation. Data were descriptively compared to a previously published pre-EMR implementation audit.

**Results:**

During the 12-month audit period, 115 patients attended prehabilitation initial assessments with an exercise clinician. EMR audit found 88.7% (*n* = 102/115) of patients were prescribed either resistance, aerobic, or both exercise types, compared to 72.4% (*n* = 84/116) pre-EMR implementation. Eighty-four percent (*n* = 97/115) of patients were prescribed an aerobic program. Of these, 91.8% (*n* = 89/97) had all required FITT exercise prescription parameters documented, compared to 28.7% (*n* = 27/94) pre-EMR implementation. Of the 80.9% (*n* = 93/115) of patients prescribed a resistance program, 89.2% (*n* = 83/93) had all exercise prescription parameters documented in the EMR compared to 0% pre-EMR implementation. For patients not prescribed exercise, 84.6% (*n* = 11/13) had valid and clearly documented reasons.

**Conclusion:**

Documentation of exercise prescription was more complete and consistent following implementation of an EMR and CRF. These findings support evidence that EMR implementation can reduce fragmentation, inefficiencies and improve consistent delivery of high-quality cancer care.

**Supplementary Information:**

The online version contains supplementary material available at https://doi.org/10.1007/s00520-026-11421-3.

## Introduction

High dose chemotherapy (HDT) followed by autologous stem cell transplant (AuSCT) is commonly used to treat several haematologic (most commonly multiple myeloma and lymphoma) and selected solid cancers [1]. In this intensive treatment, HDT aims to eliminate tumour cells, and is followed by an infusion of previously collected and cryopreserved stem-cells from the patient [1]. This results in augmented recovery of bone marrow function and hematopoiesis, allowing myeloablative doses of chemotherapy to be delivered safely [1]. While this treatment regimen results in improved cure rates in some lymphoma subtypes and progression free survival in multiple myeloma [2–4], it is also associated with a variety of adverse effects including fatigue, loss of strength, lack of appetite and nausea which can significantly impact mobility, physical activity levels and overall health-related quality of life (HRQoL) [5–7].

There is a growing body of evidence to support optimisation of patients prior to intensive cancer treatment characterised as prehabilitation [8, 9]. Prehabilitation is defined as a process on the cancer care continuum before and during cancer treatment that involves thorough screening, assessment, and treatment of physical and psychological impairments with needs-based multimodal interventions [8, 10]. For adult patients treated with AuSCT, prehabilitation has been shown to improve functional exercise capacity, improve HRQoL, reduce symptom burden, provide an earlier return to normal function, and reduce hospital length of stay [11–15].

Despite the well-known side effects and complications associated with AuSCT and the growing body of evidence supporting cancer prehabilitation, the implementation of cancer prehabilitation in Australia is modest [16]. At the participating centre a standardised multidisciplinary allied health prehabilitation service was implemented in 2019, which included targeted exercise, nutrition, and psychological interventions as standard care for patients with cancer receiving AuSCT. A previous evaluation of the prehabilitation service highlighted that exercise prescription principles (e.g., frequency, intensity, time, type [FITT]) and reasons for missing data regarding exercise interventions were not routinely documented in the patient’s file [17]. Without clear documentation of the exercise prehabilitation intervention, including FITT principles, at baseline and subsequent time points, interpretation of the service outcomes and clinical impact may not be accurate. A key recommendation from the service evaluation highlighted the importance of medical record template revision to include a case report form (CRF) for exercise including exercise prescription principles [17]. This recommendation coincided with the implementation of an electronic medical record (EMR) at the participating centre in 2020.

EMR systems have been developed and implemented in healthcare services on a widespread scale in recent years. The increasing use of EMRs has allowed for improvements in documentation structure, completeness, and quality. Evidence emphasises the importance of tailoring the EMR to the health service and ensuring clinicians receive adequate time and training to efficiently use the system [18–21]. EMR systems have been shown to improve clinical documentation, enhance workflow efficiency, and ease communication between members of the multidisciplinary team [22–26]. Currently, there is limited evidence regarding the effect of EMR implementation in allied health services, particularly with respect to documentation of exercise prescription parameters. Thus, the present study aimed to evaluate the exercise prescription documentation, including exercise prescription principles (FITT) following EMR and CRF implementation, in a prehabilitation clinical service for haematologic cancer patients receiving AuSCT. Findings were compared with those from the pre-EMR implementation evaluation [17].

## Methods

### Setting and study design

A retrospective audit of prospectively collected data from February 2023 to February 2024 of patients with cancer undergoing prehabilitation prior to AuSCT at a single centre.

### Ethics

Ethics approval was received via the quality assurance pathway from the health service’s Human Research Ethics Committee (reference number QA/67920/PMCC-2020). Clinical trial number: not applicable.

### Procedure

EMR implementation at the participating site occurred in August 2020. In 2022, an electronic CRF was developed within the EMR system by a team consisting of senior physiotherapy clinicians, senior researchers, and EMR builders. The CRF was developed to standardise documentation, enhance adherence to international exercise guidelines for people with cancer [27], and allow for accurate reporting and analysis. Aerobic training parameters required to be reported at initial assessment were frequency, intensity, and time. Resistance training parameters required at initial assessment were frequency, intensity, repetitions, and sets. The CRF was implemented in February 2023 after exercise clinicians (physiotherapists and exercise physiologists) received a one-hour training session on the new system. A documentation audit was completed one month after initiation to assess staff compliance with reporting and results were discussed with the exercise clinicians. Nil modifications were made to the CRF based on this audit.

#### Patients, referral, and enrolment

Eligibility criteria for the routinely provided prehabilitation service were as follows: all adults with cancer diagnoses, being considered for AuSCT at the participating hospital, no contraindications to exercise, and appropriate cognitive function to consent to prehabilitation and complete an independent home exercise program. Contraindications to exercise were documented in the electronic medical record by the treating haematologist. Exercise clinicians reviewed relevant notes prior to initial assessment. Most participants were undergoing induction or salvage treatment during the prehabilitation period.

#### Exercise intervention

The exercise intervention protocol for patients in this standard of care clinical service includes aerobic and resistance training prescribed at initial exercise assessment. Exercise and physical activity is individually prescribed based on initial exercise assessment findings, patient goals, and as per the Exercise and Sports Science Association guidelines, which recommend a weekly dosage of 150 min of moderate intensity aerobic exercise and two sessions of resistance exercise [28]. Aerobic exercise is either prescribed as moderate intensity continuous training or high intensity interval training based on patient assessment, safety and ability. Intensity is prescribed according to the modified-Borg scale of perceived exertion (numbered 0–10 scale), with an aim of “moderate intensity” (4–5/10) for all exercise components [29].

Patients complete exercise programs independently at home, at the hospital gym individually or in a group-based setting, in a local gym, or supervised in a community health centre. The program location was determined based on patient preference, residential location, and level of supervision needed (determined by the exercise clinician). Weekly or fortnightly telehealth or phone reviews were conducted by exercise clinicians to re-assess and progress exercise programs as appropriate for each patient. Further details of the exercise intervention are outlined as per the TIDieR guidelines [30] in supplementary file 1, updated from the previous evaluation [17].

### Outcomes

Evaluation was completed using the *Implementation* element of the Reach, Effectiveness, Adoption, Implementation, and Maintenance (RE-AIM) framework [31]. This framework was developed to plan and assess the translation of interventions into clinical practice and focuses on impact at an individual, organisation, and community level [32]. The *Implementation* component assesses the extent to which an intervention is delivered as intended in real-world settings. The analytical approach used by Crowe et al. was used as a pro forma to guide data extraction and analysis, and to facilitate comparison [17]. Data extracted from the EMR included:

1. The number of patients who were referred to the exercise component of the prehabilitation service and how many had been prescribed aerobic and resistance exercise.
2. The number of patients who had each exercise prescription parameter (FITT) documented, specifically for aerobic exercise frequency, intensity, and time and for resistance exercise frequency, intensity, sets and reps as per the developed exercise CRF.
3. Reasons for non-prescription of exercise.

### Data management and analysis

A research electronic data capture (REDCap) database was developed for collecting and storing data from clinical documentation in the hospital EMR. Descriptive statistics (patient demographics, referral statistics, and FITT reporting) were reported using counts and percentages for categorical variables and means with standard deviations, or medians with interquartile ranges (IQR) for continuous variables, dependent on distribution normality. Findings were compared descriptively to Crowe et al. (2022) [17], to determine if changes in exercise prescription documentation had occurred since the EMR and exercise CRF had been implemented. All statistical analyses were completed using both an online statistics program (JASP, version 0.95.0) and Microsoft Excel 365.

## Results

**Fig. 1:**
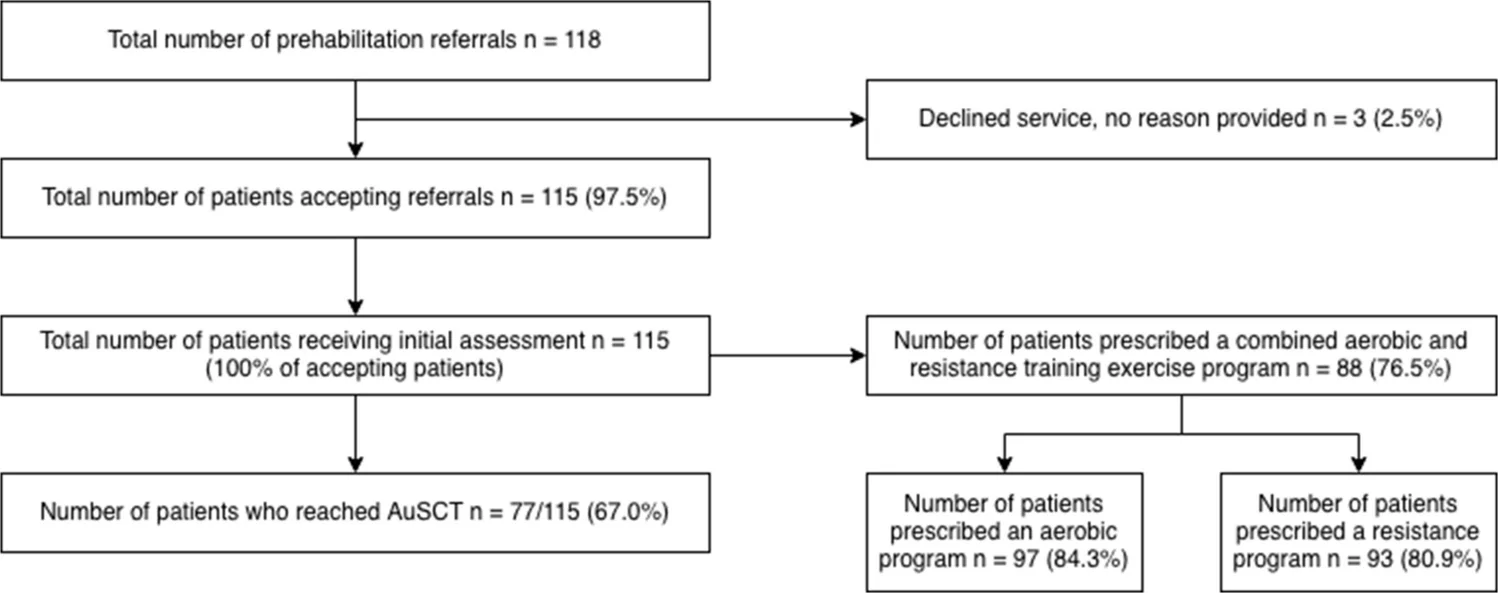
Patient flow through prehabilitation service

One hundred and eighteen eligible patients were referred to the prehabilitation service during the study period (February 2023–February 2024). Figure 1 demonstrates participant flow through the service. The majority of referred patients had been diagnosed with multiple myeloma (56.8%, *n*=67) and two-thirds resided in a major city (66.9%, *n*=79). Table 1 provides additional demographic and clinical characteristics. Following referral, three patients declined the prehabilitation service, with 115 participants completing the initial assessment.

**Table 1:** Patient characteristics

Variable	*n* = 118
Age (years), median (IQR)	61.0 (52.0 to 68.0)
Sex (male)	69 (58.5)
Cancer diagnosis	
Multiple myeloma Lymphoma Testicular cancer	67 (56.8) 49 (41.5) 2 (1.7)
Received induction/salvage anticancer treatment during prehabilitation (yes)	113 (98.2)
Rurality (MMM)	
1 2 3 4 5	79 (66.9) 11(9.3) 4 (3.4) 5 (4.2) 19 (16.1)

Values are *n* (%) unless specified. Abbreviations: *IQR*, inter-quartile range; *MMM*, rurality classified as per the Modified Monash Model classification system. MMM classifications are based on the Australian Statistical Geography Standard – Remoteness Area and classifies remoteness and population size on a scale of MM 1 to MM 7. MM 1 = major city accounting for 70% of Australia’s population and MM 7 = very remote communities [33]

### Implementation

Of the 115 patients who received an initial assessment, 88.7% (*n*=102) were prescribed an exercise program with at least one exercise type - aerobic or resistance (Table 2). Just over three-quarters (76.5%, *n*=88/115) were prescribed a combined aerobic and resistance training exercise program. By comparison, 72.4% (*n*=84) of patients were prescribed a program consisting of at least one exercise type in the pre-EMR implementation evaluation.

**Table 2:** Prescription of exercise at initial assessment and reported exercise prescription parameters per exercise type

	Exercise Type	
	Aerobic *n* (%)	Resistance *n* (%)
Combined (aerobic and resistance) programs	88 (76.5%)	
Single (aerobic or resistance) programs Total number of patients prescribed (combined or single) programs	9 (7.8%) 97 (84.3%)	5 (4.3%) 93 (80.9%)
Parameter		
Sets	N/A	92 (98.9%)
Time/Reps	95 (97.9%)	92 (98.9%)
Intensity	89 (91.8%)	83 (89.2%)
Frequency	94 (96.9%)	88 (94.6%)
All Relevant Parameters	89 (91.8%)	83 (89.2%)

**Fig. 2:**
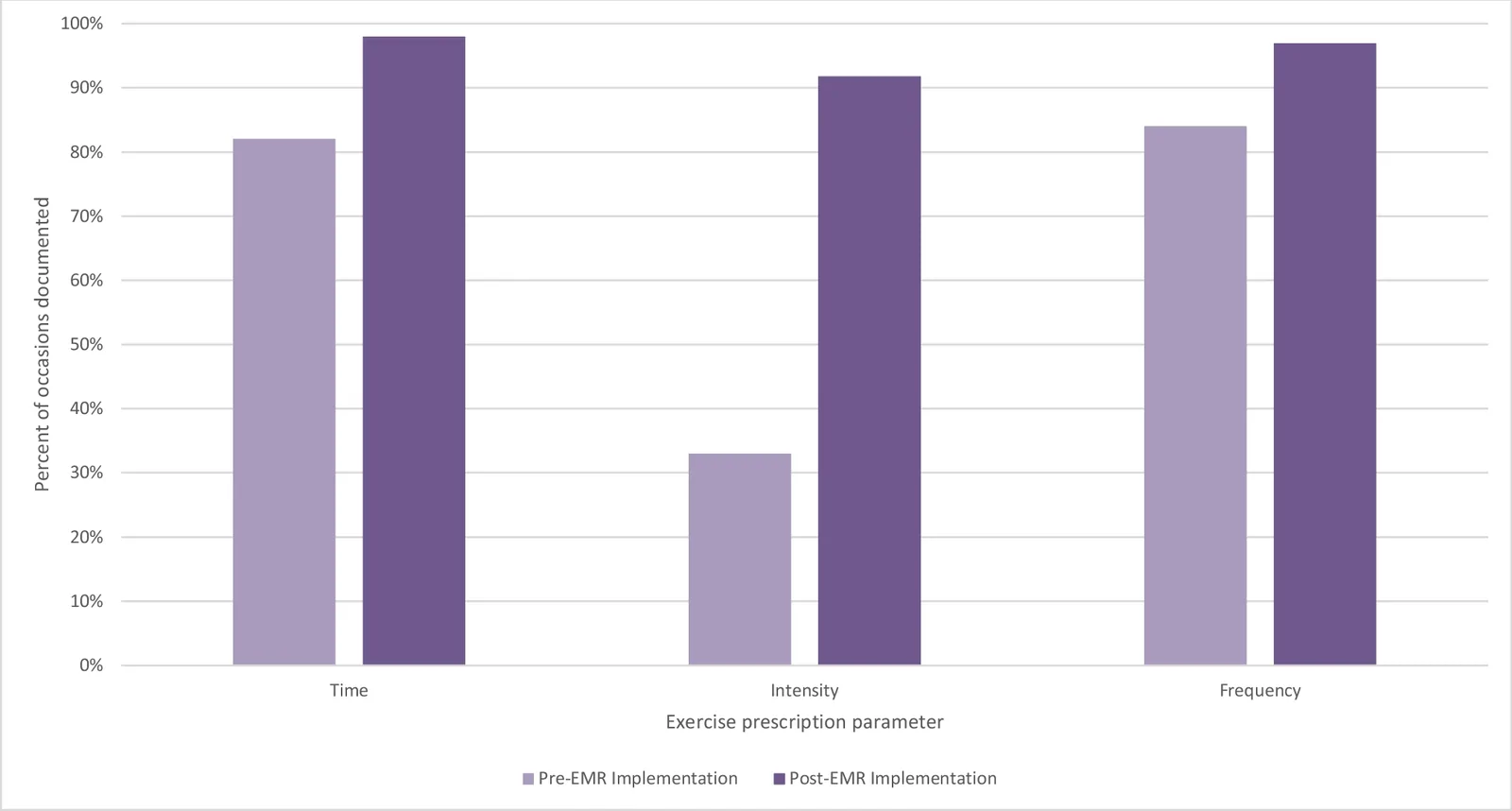
Proportions of reported FITT exercise prescription parameters for aerobic exercise

**Fig. 3:**
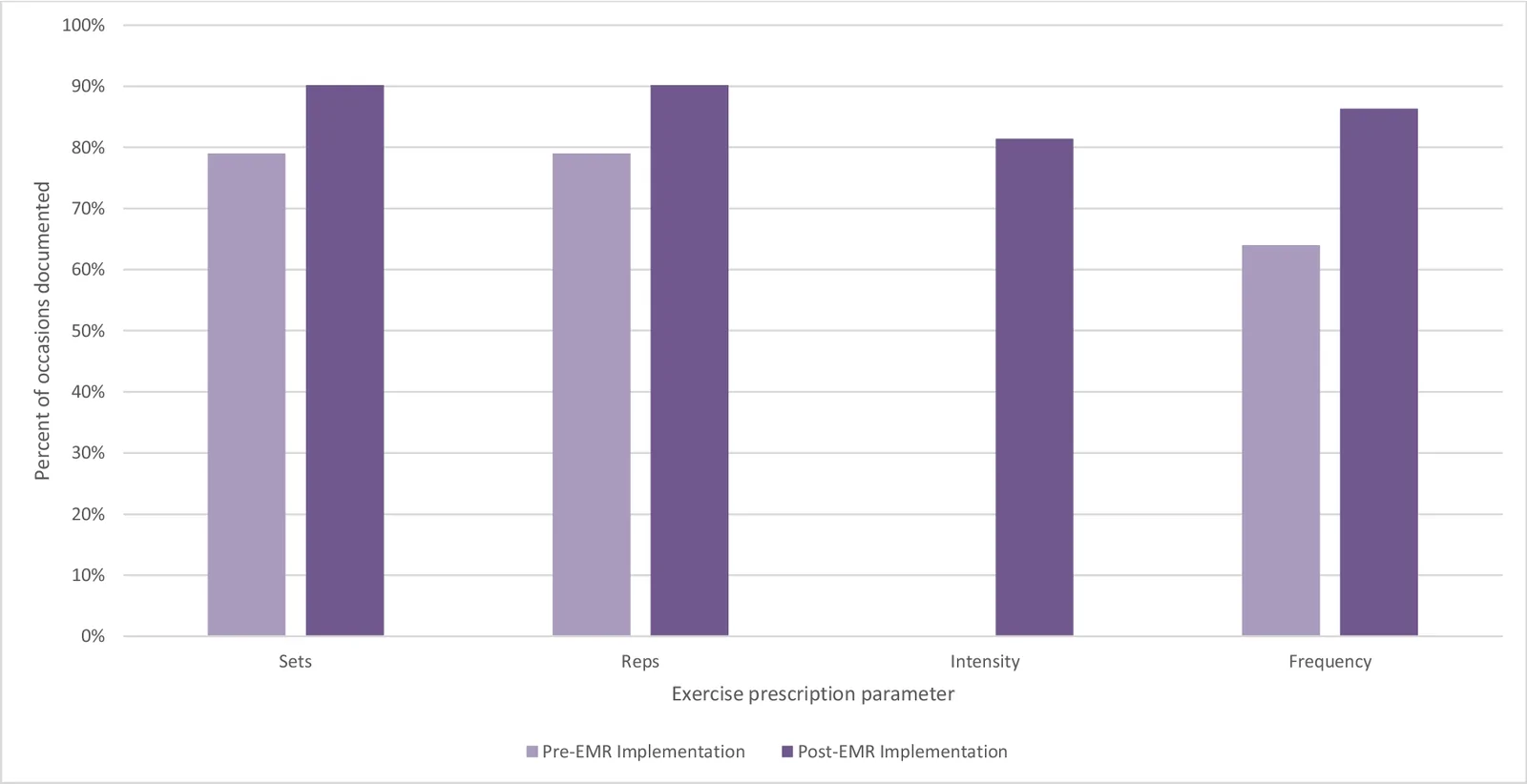
Proportions of reported exercise prescription parameters for resistance exercise

Table 2 shows the reporting of exercise parameters for the respective exercise types. Of patients who were prescribed aerobic exercise, 91.8% (*n* = 89/97) had all three required parameters of time, intensity, and frequency documented in the EMR. For the 93 patients prescribed a resistance program, 89.2% (*n* = 83/93) all four parameters of sets, repetitions, intensity, and frequency were documented in the EMR. For both types of exercise, intensity was the least reported parameter; however, reporting remained relatively high for both exercise types, at 91.8% (*n* = 89) for aerobic intensity and 89.2% (*n* = 83) for resistance intensity. Figures 2 and 3 provide comparisons of aerobic and resistance exercise parameter reporting in the pre- and post-EMR implementation evaluations. Reporting of exercise intensity was observed to change the most of all four parameters from pre-to post-EMR implementation for both exercise types; from 33.0% to 91.8% for aerobic exercise (Fig. 2) and from 0.0% to 81.4% for resistance exercise (Fig. 3).

For patients who were prescribed only one of the two exercise components (12.2%, *n* = 14/115), this was predominantly due to patients already having an established aerobic or resistance exercise program (*n* = 7) or medical contraindications (*n* = 5), outlined in Table 3. Of the 11.3% (*n* = 13) of patients who did not receive any exercise prescription, 84.6% (*n* = 11) had clear reasons documented as to why they did not have exercise prescribed; mainly already prescribed an exercise program and/or receiving prehabilitation at another service (Table 3). In the pre-EMR implementation evaluation, reasons for non-exercise prescription were not documented.

**Table 3:** Reasons for patients not being prescribed a particular exercise type

Reason	Prescribed aerobic only (n)	Prescribed resistance only (n)	Not prescribed any exercise (n)
Medical contraindication*	4	1	1
Declined a particular exercise modality	2		
Already meeting recommended exercise guidelines	3	4	4
Existing exercise program and/or receiving prehab at another service			6
No reason provided			2
Total	9	5	13

*Reasons for medical contraindications: bony metastases/bony lesion (2), deep vein thrombosis (1), pathological fracture (1), chest pain (1), having stem cells collected at time of review (1)

## Discussion

In this service audit the ‘Implementation’ element of the RE-AIM framework was used to evaluate exercise intervention parameter reporting following implementation of an EMR and exercise CRF. The results demonstrate that reporting of FITT exercise parameters was high, with over 90% of patients who received an aerobic exercise program, and over 85% receiving resistance exercises, having all relevant FITT exercise parameters recorded. Reasons for non-exercise prescription were very clearly documented for 85% of patients in the post-EMR implementation audit. Where patients were prescribed only one exercise type the reasons for this were documented for all patients. The documented reasons for non-exercise prescription were valid and commonly a result of patients already meeting recommended levels of exercise or medical contraindications to exercise in an oncology population. Based on these findings, implementation of the EMR and exercise CRF into the health service has been successful.

Post-EMR implementation, the level of exercise prescription reporting demonstrated a clear improvement from the pre-EMR implementation findings (2019–2020) reported by Crowe et al. [17]. In the current study, the reporting of all FITT parameters for both aerobic and resistance exercise increased post-EMR and exercise CRF implementation, enabling more robust evaluation of how closely routine clinical practice aligns with established, evidence-based exercise guidelines for people living with cancer [27, 34]. Notably, the largest improvements were observed in documentation of aerobic and resistance exercise intensity prescribed. This finding is clinically meaningful, as the effectiveness of cancer exercise interventions on patient-reported and objectively measured outcomes such as fatigue, health-related quality of life, cardiovascular fitness and physical function is closely linked to the prescription of at least moderate-intensity exercise [35]. Furthermore, emerging evidence suggests that higher-intensity approaches, including high-intensity interval training (HIIT), can result in greater improvements in cardiovascular fitness compared to moderate-intensity continuous training (MICT) [36]. Improved intensity reporting in our study therefore represents an important step toward enabling exercise clinicians to prescribe, monitor and evaluate exercise at intensities consistent with the evidence.

The improvements observed in our study are consistent with previous findings across medical and allied health literature, which demonstrate that EMR implementation is associated with more structured, standardised, and comprehensive clinical documentation [23–26]. In all four studies the implementation of an EMR resulted in improvements to documentation standardisation and structure, as well as better alignment with clinical assessments. For example, Russell & McNeill (2022) found that utilising EMRs resulted in a 130% increase in the production of individualised care plans as well as faster plan initiation for patients in a hospital environment, outlining how EMRs can improve the volume and quality of patient-centred documentation. Similarly, Feely et al. (2023) found that allied health professionals report increased confidence and satisfaction with documentation following the implementation of an EMR [23]. Our findings extend this literature by demonstrating that EMR-enabled standardisation, through use of a CRF, can specifically enhance the quality of exercise prescription documentation, an area critical for high-quality cancer care.

Beyond documentation quality, EMRs are frequently implemented with the intention of improving interdisciplinary co-ordination and workflow efficiency. Consistent with previous reports, the introduction of an EMR in our study reduced fragmentation, inefficiencies and potential data errors in exercise prescription documentation, which had previously been entered in duplicate by exercise clinicians in multiple systems — the medical record and an exercise prescription software ‘SimpleSet’. SimpleSet exercise programs can be copied and pasted into the EMR without duplication, thus reducing inefficiencies. This mirrors findings of prior studies which identified that the introduction of an EMR improved the quality of care delivered to patients by enhancing workflow efficiency [22, 24, 25]. Merrell et al., (2021) found that structured templates within an EMR resulted in a reduction in redundant documentation. Cashman et al. (2021) demonstrated that moving from paper-based documentation to an EMR allowed for information to be collated together more effectively, particularly across services and sites. However, it appears that EMRs do not always improve efficiency. Feely et al. (2023) observed a 5.4% increase in documentation time following EMR implementation, which may reflect the demand of learning the new system along with variable digital literacy skills. These mixed findings emphasise the importance of adequate training, as provided in our study, and ongoing system refinements to maximise the benefits of EMRs.

The most commonly reported benefit of EMR use in the broader healthcare literature and within hospital settings is improved patient safety, particular through reductions in medication errors and thus fewer adverse drug events [18–21]. A further finding of the effects of EMR on patient care is an improvement in adherence to evidence-based clinical guidelines [18, 19, 21]. Adhering to clinical guidelines mitigates practice variability and reduces medical errors, which can have secondary effects on improving patient outcomes [19]. While our study did not focus on medication safety, a similar mechanism may underpin the observed improvement in exercise prescription quality. We observed a small increase in the proportion of patients who were prescribed a combined aerobic and resistance exercise program, from 72.4% pre- to 76.5% post-EMR implementation. This aligns with recommendations in international clinical guidelines for exercise prescription in oncology and supports more consistent delivery of evidence-based practice care [27, 28]. Taken together, these findings suggest that EMRs can play a critical enabling role in implementing clinical guidelines into routine practice, thereby supporting higher-quality, safer and standardised care for patients undergoing intensive cancer therapies.

While the present study has provided several insights into the effect of EMR implementation on exercise prescription documentation, it is not without limitations. Limitations include the absence of qualitative data to understand the experiences of exercise clinicians working within the prehabilitation service prior to and following implementation of the EMR and exercise CRF. Additionally, while the audit has included an evaluation of the fidelity of exercise prescription delivery, we did not evaluate whether patients adhered to performing their exercise programs as prescribed.

Clear directions for future research can be determined from the reported findings. Further research into the effectiveness of prehabilitation for AuSCT patients as evaluated by functional capacity, HRQoL, and hospital length of stay is needed to support the resourcing required for the development of future prehabilitation programs. Additionally, this audit did not specifically evaluate the time taken to complete the EMR and CRF documentation, clinician digital literacy confidence, nor the impact on patient outcomes. The inclusion of measures of efficiency as well as clinician acceptability and patient outcomes may be useful in future research.

## Conclusion

This audit found that introduction of an EMR with a tailored exercise CRF resulted in substantial improvements in the completeness, consistency, and transparency of exercise prescription documentation within a cancer prehabilitation service. Additionally, there was improved reporting of valid reasons for not prescribing exercise, reflecting decision-making in line with international clinical guidelines. These findings are consistent with broader medical and allied health literature demonstrating that EMR-enabled standardisation improves documentation quality, workflow efficiency, and guideline adherence, while reducing fragmentation and duplication across systems. At a local level, current documentation allows evaluation of prescribed exercise programs and FITT parameters against clinical practice guidelines, supporting evidence-based patient care. Furthermore, findings could drive exercise clinician adherence to meet clinical service protocols. Finally, the study findings can inform health services of the benefits of implementing a well-structured EMR, including a tailored exercise CRF.

## Supplementary Information

Below is the link to the electronic supplementary material.

ESM 1 (DOCX 74.1 KB)

## Data Availability

The datasets used and/or analysed during the current study are available from the corresponding author on reasonable request.
