# Supplementary material for: The effects of implementing an electronic medical record on prehabilitation exercise prescription reporting for autologous stem cell transplantation
Source: Support Care Cancer. 2026 Nov 6;34(12):1199. doi: 10.1007/s00520-026-11421-3 (PMC13739943; doi:10.1007/s00520-026-11421-3)
Supplement: Supplementary file 1 [file 520_2026_11421_MOESM1_ESM.docx]

**Supplementary Files**

**The effects of implementing an electronic medical record on prehabilitation exercise prescription reporting for people with haematological cancer**

**Supportive Care In Cancer**

Jess Crowe, Shaza Abo, Linda Denehy, Amit Khot, Trish Joyce, Bernd Faveere, Samantha Hui Bon Hoa, Lucas Elias, Michelle Flechas, Marilyn Webster, Lara Edbrooke

Corresponding author: A/Prof Lara Edbrooke - Department of Physiotherapy, The University of Melbourne**;** Centre for Health Services Research in Cancer, Peter MacCallum Cancer Centre, Melbourne; The Sir Peter MacCallum Department of Oncology.

**Supplementary File 1**

**Template for Intervention Description and Replication (TIDieR)**

**Item 1. Brief Name**

Multidisciplinary allied health (exercise, nutrition, and psychology) prehabilitation for haematologic cancer patients being considered for intensive chemotherapy with autologous stem cell transplant (AuSCT) support. The service is multidisciplinary; however, only the exercise component is relevant for this study and will be detailed in this checklist.

**Item 2. Why: Describe any rationale, theory, or goal of the elements essential to the intervention.**

Cancer prehabilitation is defined as a process on the care continuum prior to major oncological treatment that involves thorough screening, assessment, and treatment of physical impairments with targeted interventions [1]. The aim of cancer prehabilitation is to reduce post-treatment complications and enhance functional capacity [2]. Exercise prehabilitation involves strength and aerobic exercise for patients prior to a major procedure, in this case, an AuSCT, with the goal of improving physical function to enhance post-procedure recovery. There is growing evidence for exercise prehabilitation prior to stem cell transplant for haematologic cancer[3-5].

**Item 3. What (materials): describe any physical or informational materials used in the intervention including those provided to participants or used in intervention delivery or in training of intervention providers**

**Exercise materials:** Patients were provided a printed copy of their exercise program at the initial assessment using physiotherapy exercise prescription software, SimpleSet [6]. Resistance bands were provided if patients did not have access to weights for resistance training.

**Item 4. What (procedures): describe each of the procedures, activities, and/or processes used in the intervention, including any enabling or support activities**

**Patient referral and enrolment:** Patients were referred for allied health services and screened during a telephone call from the Allied Health Assistant for exercise program eligibility.

**Following screening:** Eligible patients were invited to participate in the service, which, as they were informed, was now routine care. Upon acceptance patients were booked for individual assessments with the exercise clinician.

**Outcome measures:** Objective outcomes measuring functional exercise capacity and strength were completed at initial and pre-transplant exercise assessment as described below. These assessment findings were used to provide Individualised exercise prescription and tracked patient performance over time. Tests were standardised and treating clinicians received verbal and written education on conduct of each outcome individually prior to service commencement.

The six-minute walk test (6MWT) is a commonly used submaximal test of functional exercise capacity and is valid and reliable in people with cancer[7, 8]. The 6MWT was completed using a 30-metre corridor and standardised encouragement. The Baseline Lite Hydraulic handgrip dynamometer (Fabrication Enterprises Inc.) was used to assess handgrip strength and data from this device correlates with the Jamar dynamometer [9], which has demonstrated validity and reliability in the oncology population[10]. The validated 30-second sit-to-stand test (30secSTS), performed according to standard protocols, was used to assess lower limb strength and function [11].

**Exercise Physiology/Physiotherapy:** An individually tailored exercise program consisting of cardiovascular continuous moderate intensity aerobic and resistance exercise was prescribed at initial exercise assessment. The exercise intervention was based upon initial assessment findings and aimed to progress towards meeting the Exercise and Sports Science Australia [12] and American College of Sports Medicine [13] recommendations of 150 minutes of moderate-intensity aerobic exercise and two resistance exercise sessions per week. Aerobic training modality (bike, walking, treadmill) was prescribed according to patient preference. Patients were instructed to monitor intensity via the modified Borg scale (aiming for a moderate-high intensity ‘4- 5 – somewhat hard'). Resistance exercise prescribed involved functional exercises which often included: sit-to-stand, heel-raises, squats, step-ups, bench push-ups, bicep curls, overhead press, shoulder abduction. Exercises were prescribed based on 10 repetition maximum (10RM) and then each exercise started at 80% of this, with patients performing two sets of 8-10 reps.

Patients completed the program either independently at home, in a local gym, supervised at a community health centre, or at the hospital gym. The program location was determined by patient residential location, preference, and the level of supervision needed.

Patients who were deemed moderate – high risk (6-minute walk test (6MWT) distance

<540 m) were encouraged to attend at least 2 supervised exercise sessions per week, whilst continuing their individualised home exercise program on other days. These sessions ran face-to-face in the hospital gym two times a week or virtually three times a week.

Face-to-face classes were supervised by the exercise clinician; patients exercised using a treadmill or cycle for aerobic exercise and completed an individually tailored resistance program. Virtual classes were run by exercise clinicians and included aerobic and resistance-based circuit training. Circuits were tailored to individuals based on exercise tolerance and patient symptoms. Patients who would benefit from supervised exercise but were unable to attend the hospital gym due to location were referred to community exercise clinicians to complete their prescribed program, with the recommendation to attend the community service at least twice a week. Patients with reported or perceived lack of motivation were referred to the psychology team if consenting. Home based exercise programs were printed or emailed to patients at initial assessment.

**Item 5. Who Provided: for each category of intervention provider (for example, psychologist, nursing assistant), describe their expertise, background and any specific training given.**

Staff delivering the service were physiotherapists or exercise physiologists with an average of three years specialised oncology experience.

**Item 6. How: Describe the modes of delivery (such as face to face or by some other mechanism, such as internet or telephone) of the intervention and whether it was provided individually or in a group.**

**Exercise Physiology/Physiotherapy**: In 2020, PMCC transitioned to a hybrid face-to- face and telehealth model of care due to the COVID-19 pandemic. Since then, telehealth has become a more prominent feature of all care due to convenience for patients and improved use/engagement among both clinicians and patients. Thus, the COACH service now implements a hybrid style of care including in-person and virtual telehealth appointments, whilst preferencing face-to-face initial assessments where feasible.

An initial individual face-to-face (at the hospital) or virtual telehealth consultation was conducted and treatment initiated. If the first appointment was virtual, initial outcome measures would be conducted at the next face-to-face appointment, ideally the second

appointment. If deemed appropriate, a 30-second sit-to-stand was conducted at the

initial telehealth appointment. The 30-second sit-to-stand test has been deemed feasible and safe to complete via telehealth[14]. Patients completed the prescribed exercise intervention either independently at home, in a local gym, supervised at a community health centre or at the hospital gym, or supervised virtually via telehealth. Patients who were deemed moderate – high risk (6MWT distance <540 m) were encouraged to attend at least 2 supervised exercise sessions per week, whilst continuing their individualised home exercise program on other days. These sessions ran face-to-face in the hospital gym two times a week or virtually three times a week.

Weekly, fortnightly, or monthly telehealth (via videoconferencing), phone, or face-to- face follow-up progress review was planned for each patient if not attending weekly group supervised sessions. Frequency of follow-up review was determined by initial assessment; patients with poorer exercise capacity were followed up weekly (6MWT distance <440 m), moderate risk fortnightly (6MWT distance 440 m – 540 m) or monthly (6MWT distance >540 m)

**Item 7. Where: Describe the type(s) of location(s) where the intervention occurred including any necessary infrastructure or relevant features.**

**Exercise Physiology/Physiotherapy**: Initial consultation occurred either in the hospital gym or via telehealth. Patients completed the prescribed exercise intervention either independently at home, in a local gym, supervised at a community health centre, supervised via telehealth at home, or at the hospital gym. Weekly or fortnightly follow up occurred at the hospital gym or via phone or telehealth (videoconferencing). If follow up was via phone or telehealth this intervention occurred anywhere the patient had internet or phone access and an appropriate device (predominantly in the home environment).

**Item 8. When and how much: Describe the number of times the intervention was delivered and over what period of time including the number of sessions, their schedule and their duration, intensity or dose.**

Prehabilitation programs were of 4-12 weeks duration depending on the timing of referral in relation to medical treatment interventions (autologous stem cell transplant).

**Exercise Physiology/Physiotherapy**: The initial consultation was of 60 minutes' duration. Patients who were deemed moderate to high risk were encouraged to attend at least two of the five 60-minute supervised group sessions offered weekly (either face-to-face in the hospital gym or via telehealth) and complete additional sessions (if any) independently with their individualised home exercise program. Patients who did not attend supervised exercise and were completing their program at home were recommended to perform a minimum of two resistance and three aerobic exercise sessions a week as per the Exercise Guidelines for Cancer Survivors [13]. Patients' exercise programs were reviewed weekly – monthly for the duration of the prehabilitation program and were individually progressed as outlined in Item 9 below. Follow up appointments were scheduled for 30 minutes.

**Item 9. Tailoring: If the intervention was planned to be personalised, titrated or adapted, then describe what, why, when and how.**

**Exercise Physiology/Physiotherapy:** Program adherence was monitored using patient self-reporting at follow up appointments. When patients reported improvements at follow-up appointments, resulting in exercises being performed at less than moderate intensity, the exercise parameters were progressed to maintain moderate intensity. If patients were unable to complete the prescribed program at moderate intensity, exercise parameters were reduced based on individual requirements. E.g.:

1. Progression of aerobic exercise program: ensure continuing to work at > Borg 3, increase aerobic duration, increase aerobic speed. If on bike can also increase resistance provided to ensure still achieving Borg 4.
2. Progression of strengthening exercise program; Increase until patient can perform three sets of 15 then increase weight/reduce chair seat height and drop back sets and reps to original number, gradually increase back to two sets of 15 then progress to three sets and repeat.

**Item 10. Modifications.**

The intervention was not modified. However, findings of this study will be used to modify the ongoing program.

**Item 11. How well (planned): if intervention adherence or fidelity was assessed, describe how and by whom, and if any strategies were used to maintain or improve fidelity, describe them.**

**Training of providers:** Staff were informed about the service during scheduled staff meetings. The program model of care was distributed, and outcome measure guidance provided to staff delivering the service via a procedural training manual. These were based on guidelines for exercise interventions and standardised measurement of specific outcomes using valid measures. Senior prehabilitation staff participated in weekly staff meetings during the first 6 months of service commencement where service challenges including assessment and intervention fidelity were discussed and processes refined. These meetings became monthly for the following 6 months, and then bi-monthly thereafter.

**Delivery of intervention:** An analysis of exercise intervention fidelity was conducted. Results of this analysis in conjunction with the implementation of electronic medical records (EMR) at the hospital in August 2020 led to changes in exercise prescription reporting and staff training.

**Receipt of intervention**: Adherence to the prescribed exercise intervention was monitored using patient self-report at follow up assessment.

**Item 12. How well (actual): if intervention adherence or fidelity was assessed, describe the extent to which the programme was delivered as planned.**

Exercise treatment fidelity was not evaluated for the current study. This study investigated the proportion of patients who received the exercise intervention (as documented in clinical notes) and assess whether the exercise parameters were documented by the clinician in the patient's medical record based upon the frequency, intensity, time and type (FITT) exercise principles.

**References**

1.	Silver JK, Baima J. Cancer prehabilitation: an opportunity to decrease treatment-related morbidity, increase cancer treatment options, and improve physical and psychological health outcomes. American Journal of Physical Medicine & Rehabilitation. 2013;92(8):715-27.

2.	Macmillan Cancer Support. Prehabilitation for people with cancer 2019.

3.	Coleman EA, Hall-Barrow J, Coon S, Stewart CB. Facilitating exercise adherence for patients with multiple myeloma. Clinical Journal of Oncology Nursing 2003 Oct;7(5):529-534. 2003.

4.	Van Haren IEPM, Staal JB, Potting CM, Atsma F, Hoogeboom TJ, Blijlevens NMA, et al. Physical exercise prior to hematopoietic stem cell transplantation: A feasibility study. Physiotherapy Theory and Practice. 2018;34(10):747-56.

5.	Wood WA, Phillips B, Smith-Ryan AE, Wilson D, Deal AM, Bailey C, et al. Personalized home-based interval exercise training may improve cardiorespiratory fitness in cancer patients preparing to undergo hematopoietic cell transplantation. Bone Marrow Transplantation. 2016;51(7):967-72.

6.	SimpleSet. SimpleSet 2009 [Available from: <https://simpleset.net>.

7.	Holland AE, Spruit MA, Troosters T, Puhan MA, Pepin V, Saey D, et al. An official European Respiratory Society/American Thoracic Society technical standard: field walking tests in chronic respiratory disease. Eur Respir J. 2014;44(6):1428-46.

8.	Schmidt K, Vogt L, Thiel C, Jäger E, Banzer W. Validity of the six-minute walk test in cancer patients. International Journal of Sports Medicine. 2013;34(7):631-6.

9.	Mathiowetz V, Kashman N, Volland G, Weber K, Dowe M, Rogers S. Grip and pinch strength: normative data for adults. Archives of Physical Medicine and Rehabilitation. 1985;66(2):69-74.

10.	Trutschnigg B, Kilgour RD, Reinglas J, Rosenthall L, Hornby L, Morais JA, et al. Precision and reliability of strength (Jamar vs. Biodex handgrip) and body composition (dual-energy X-ray absorptiometry vs. bioimpedance analysis) measurements in advanced cancer patients. Applied Physiology, Nutrition, and Metabolism. 2008;33(6):1232-9.

11.	Jones CJ, Rikli RE, Beam WC. A 30-s chair-stand test as a measure of lower body strength in community-residing older adults. Research Quarterly for Exercise and Sport. 1999;70(2):113-9.

12.	Hayes SC, Newton RU, Spence RR, Galvão DA. The Exercise and Sports Science Australia position statement: exercise medicine in cancer management. Journal of Science and Medicine in Sport. 2019;22(11):1175-99.

13.	Campbell KL, Winters-Stone KM, Wiskemann J, May AM, Schwartz AL, Courneya KS, et al. Exercise Guidelines for Cancer Survivors: Consensus Statement from International Multidisciplinary Roundtable. Med Sci Sports Exerc. 2019;51(11):2375-90.

14.	Bowman A, Denehy L, Benjemaa A, Crowe J, Bruns E, Hall T, et al. Feasibility and safety of the 30-second sit-to-stand test delivered via telehealth: An observational study. Pm R. 2023;15(1):31-40.
